# Transcriptome analyses of the *Dof*-like gene family in grapevine reveal its involvement in berry, flower and seed development

**DOI:** 10.1038/hortres.2016.42

**Published:** 2016-08-31

**Authors:** Danielle Costenaro da Silva, Vítor da Silveira Falavigna, Marianna Fasoli, Vanessa Buffon, Diogo Denardi Porto, Georgios Joannis Pappas, Mario Pezzotti, Giancarlo Pasquali, Luís Fernando Revers

**Affiliations:** 1 Graduate Program in Cell and Molecular Biology, Center for Biotechnology, Universidade Federal do Rio Grande do Sul, Porto Alegre, RS 91501-970, Brazil; 2 Dipartimento di Biotecnologie, Università degli Studi di Verona, Verona 37134, Italy; 3 Embrapa Uva e Vinho, Bento Gonçalves, RS 95701-008, Brazil; 4 Embrapa Semiárido, Petrolina, PE 56302-970, Brazil; 5 Department of Cell Biology, Universidade de Brasília, Brasília, DF 70910-900, Brazil

## Abstract

The Dof (DNA-binding with one finger) protein family spans a group of plant transcription factors involved in the regulation of several functions, such as plant responses to stress, hormones and light, phytochrome signaling and seed germination. Here we describe the *Dof*-like gene family in grapevine (*Vitis vinifera* L.), which consists of 25 genes coding for Dof. An extensive *in silico* characterization of the *VviDofL* gene family was performed. Additionally, the expression of the entire gene family was assessed in 54 grapevine tissues and organs using an integrated approach with microarray (cv Corvina) and real-time PCR (cv Pinot Noir) analyses. The phylogenetic analysis comparing grapevine sequences with those of Arabidopsis, tomato, poplar and already described *Dof* genes in other species allowed us to identify several duplicated genes. The diversification of grapevine *DofL* genes during evolution likely resulted in a broader range of biological roles. Furthermore, distinct expression patterns were identified between samples analyzed, corroborating such hypothesis. Our expression results indicate that several *VviDofL* genes perform their functional roles mainly during flower, berry and seed development, highlighting their importance for grapevine growth and production. The identification of similar expression profiles between both approaches strongly suggests that these genes have important regulatory roles that are evolutionally conserved between grapevine cvs Corvina and Pinot Noir.

## Introduction

Dof (DNA-binding with one finger) proteins are plant exclusive transcription factors characterized by a conserved 52 amino acid segment encompassing a C_2_C_2_-type zinc finger.^1^ The Dof domain is usually found in the N-terminal region of these proteins, specifically binding AAAG sequences in gene promoters. The C-terminal region is responsible to interact with other regulatory proteins in order to activate gene expression (reviewed in Yanagisawa^1^ and Hir and Bellini^2^). In contrast to the highly conserved DNA-binding domain, sequences outside the Dof domain diverge widely, suggesting that Dof proteins are regulating several biological functions. Indeed, functional studies conducted so far confirm this assumption, demonstrating Dof involvement in seed germination,^3–9^ seed development,^10–14^ flowering control,^15–18^ flower abscission,^19^ pollen development,^20^ among many other functions (recently reviewed in Gupta *et al.,*^21^ Hir and Bellini^2^ and Noguero *et al.*^22^).

After the first isolation of a complementary DNA from maize encoding a Dof domain-containing protein, these transcription factors have been identified in a variety of angiosperms including Arabidopsis, barley, pea, potato, pumpkin, rice, tobacco and wheat.^1^ Thereafter, *Dof*-like genes were identified in several other species such as grapevine,^23^ *Chlamydomonas reinhardtii*, *Jatropha curcas*, maritime pine, pea, the moss *Physcomitrella patens*, sorghum, soybean and sweet potato.^21,22^ In terms of family composition, the first genome-wide report for *Dof* genes was for Arabidopsis, which presented 36 Dof-encoding genes and one pseudogene.^1^ Thereafter, many studies identifying *Dof* genes have been reported, with 30 members in rice,^24^ 41 in poplar,^25^ 26 in barley,^26^ 19 in moss,^27^ 12 in the fern *Selaginella moellendorffiii*,^27^ 10 in loblolly pine,^27^ 31 in wheat,^28^ 27 in *Brachypodium distachyon*,^29^ 10 in pine,^30^ 34 in tomato,^31^ 78 in soybean,^32^ 25 in sugarcane,^33^ 76 in Chinese cabbage,^34^ 46 in maize,^35^ 35 in potato^36^ and 46 in carrot.^37^

We have previously conducted a representational difference analysis aiming to identify genes related to seedlessness during specific developmental stages in grapevine.^23^ Among the expressed sequence tags identified, a putative *Dof* gene was isolated in the developmental stage of four-weeks after fruit set. We therefore decided to further investigate the presence of other genes in the grapevine genome potentially encoding Dof proteins. Here we demonstrate that 25 *Dof*-like genes are present in grapevine (*VviDofL*). *In silico* analyses, including a phylogenetic approach that revealed clusters of paralogous and orthologous genes, allowed the prediction of putative functions for these genes. Finally, the expression profiles of all 25 *VviDofL* genes were established by whole-transcriptome data and strongly suggested the involvement of some of these genes in the regulation of berry, flower and seed development.

## Material and methods

### Bioinformatic analysis of nucleotide and amino acid sequences

A VviDofL sequence previously identified by the group^23^ was used as query in a genome-wide search for putative Dofs using the *BLASTP* tool^38^ implemented in the 12X Grape Genome Browser V1 (http://genomes.cribi.unipd.it/),^39,40^ which was verified in the V2 version with the same 25 VviDofL. In order to confirm the results, the Dof-seed sequence (accession number PF02701) available in the Pfam database^41^ was used to perform a HMM search using Hmmer v3.0 (http://hmmer.org/) to find predicted grapevine proteins in the 12X Grape Genome Browser V1. All predicted gene models identified by both strategies were compared with the Pfam database^41^ in order to confirm the presence of the Dof domain (PF02701), and the ones without the characteristic domain of the gene family were excluded from further analyses. The chromosomal locations of *VviDofL* genes were obtained at the Grape Genome Browser, whereas marker locations were obtained at the National Center for Biotechnology Information database (http://www.ncbi.nlm.nih.gov/). All data were compiled into a figure using the *MapChart v.2.2* (Wageningen, Netherlands) software.^42^

Deduced amino acid sequences of all grapevine DofL proteins were searched for the presence of conserved domains using MEME Suite v.4.9.0.^43^ Default parameters were used with the following exceptions: the motif distribution among sequences was set to any number of repetitions; the maximum number of motifs was set to 15; the maximum motif width was defined between 10 and 52 amino acids; and an *e*-value cutoff of 1^−10^ was adopted. All identified motifs were annotated by comparison with conserved motifs in Pfam^41^ and SMART^44^ databases. Protein subcellular localization was predicted using the Plant-mPLoc database (http://www.csbio.sjtu.edu.cn/bioinf/plant-multi/#).^45^ Full-length protein sequences of grapevine, Arabidopsis, poplar, tomato and other already described Dof-encoding genes (searched in March, 2016) were aligned using the ClustalW software.^46^ Accession numbers are shown in Supplementary Table S1. Two phylogenetic trees were inferred using MrBayes v.3.2.6 software^47^ employing the mixed amino acid substitution model in the default settings. The first tree performed a 14.81 million generation run, sampled every 100 generations and the first 25% trees were discarded as burn-in. The remaining ones were summarized in a consensus tree. The alignment of the second tree was cured to eliminate poor alignment positions and divergent regions using Gblocks 0.91b (Barcelona, Spain) software in default settings, except gap positions that were set to half.^48^ The cured alignment was composed of 50 amino acids spanning the Dof domain region. The tree was analyzed as previously described but using 9.2 million generation run. Phylogenetic trees were visualized and edited using FigTree v.1.4 (http://tree.bio.ed.ac.uk/software/figtree/).

### Analysis of whole *VviDofL* transcriptome

In order to fully characterize the expression of *VviDofL* genes during grapevine development, 54 tissues and organs of *Vitis vinifera* cv Corvina (clone 48, rootstock 41B) were analyzed using a comprehensive genome microarray. Plant materials used for the construction of the database encompassed all major grapevine organs in different developmental stages, and included data from berries during post-harvest withering, green and woody tissues, as well as specialized tissues such as pollen. Genome-wide transcriptome analysis was performed by microarray as described.^49^ The expression data was analyzed by hierarchical clustering on the whole 54-sample data set. Pearson’s correlation distance was used as the metric, and T-MeV v4.81 (Boston, MA, USA) software was used to create the transcriptional profiles dendrograms for both genes and samples.^50^ Expression data are shown as normalized based on the mean center genes/rows adjustment.

### Real-time PCR

Grapevine (*Vitis vinifera* L. cv Pinot Noir grafted on Paulsen 1103) samples from at least seven independent plants located in experimental plots at Embrapa Uva e Vinho (Bento Gonçalves/RS, Brazil) were sampled during the 2008/2009 growing season. Three biological replicates consisted of tissues from three independent plants kept separately throughout the analysis. The developmental stages were defined as described by Coombe^51^ (modified E-L system) except when not indicated: roots of *in vitro* cultivated plants, 5 cm diameter leaves, tips of 10 cm stems (E-L 12), inflorescences and tendrils from pre-anthesis (E-L 17), summer buds (E-L 31), 7 mm-large berries (E-L 31), pre-véraison and véraison berries (E-L 34–35). Pre-véraison and véraison berries were sampled from the same bunch by separating half-colored berries from green colored berries. All samples were frozen in liquid nitrogen in the field and stored at −80 °C until RNA extraction.

Primer pairs were designed using the Primer3 program (http://frodo.wi.mit.edu/primer3, Supplementary Table S2). Total RNA was extracted from frozen tissues using the Purelink RNA Reagent (Invitrogen, Carlsbad, CA, USA) and protocols recommended by the manufacturer. All RNA preparations were treated with DNAse (Fermentas, Waltham, MA, USA) in order to eliminate residual DNA contamination. Quantity and quality of total RNAs were evaluated with the Qubit Quantitation Platform (Invitrogen) and standard 1.5% agarose gel electrophoresis in 1× MOPS buffer. Complementary DNA was prepared using M-MLV Reverse Transcriptase (Promega, Madison, WI, USA). Transcript levels were determined by real-time PCR using a StepOne Plus PCR system (Applied Biosystems, Foster City, CA, USA) and SYBR Green (Applied Biosystems). The cycling protocol consisted of one step at 95 °C for 5 min, followed by 40 cycles at 95 °C for 15 s and 60 °C for 35 s, and finished by a dissociation curve between 60 and 95 °C. The specificity of PCR amplifications was assessed by the presence of a single peak in the melting curves. Biological samples (*n*=3) were analyzed in three technical replicates. Glyceraldehyde 3-phosphate dehydrogenase and tubulin were used as reference genes^52^ using the 2−∆∆Ct method.^53^

## Results

### Identification and annotation of grapevine *DofL* genes

**Figure 1:**
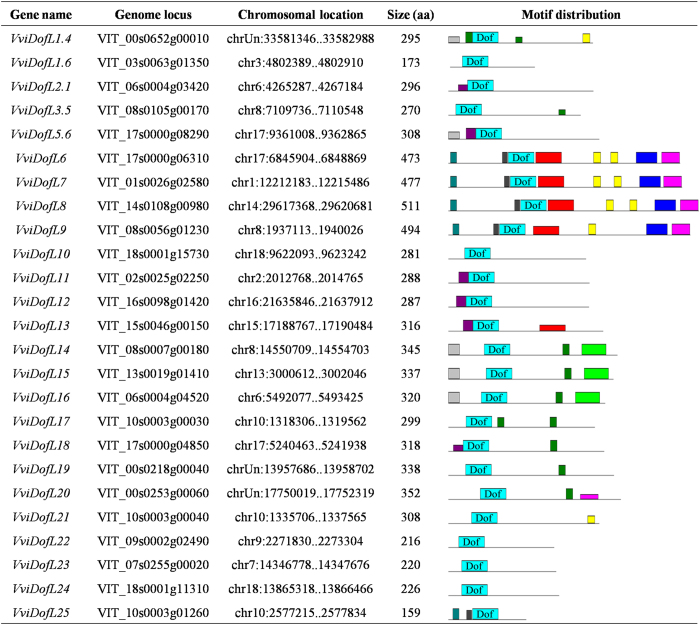
Identification of grapevine *DofL* genes. Genome locus is provided by the ‘Grape Genome’ (http://genomes.cribi.unipd.it/). Chromosomal localization and deduced peptide lengths are shown. Schematic view of the conserved motifs between *VviDofL*-deduced protein sequences was performed by the *MEME* suite.^43^ Colored boxes represent conserved motifs (Supplementary Figure S3). The height of the motif box is proportional to −log (*P* value), with the maximum height denoting a *P* value of e^−10^. Gray lines represent non-conserved sequences. *VviDofL18* was previously named *VvDof1* by Costenaro-da-Silva *et al.*^23^

In order to identify putative Dof proteins in grapevine, a VviDofL sequence previously identified by the group^23^ was used as query in *BLAST* searches, resulting in the identification of 25 genes possibly coding for Dof proteins. This result was further confirmed in a HMM search using the Dof-seed sequence available at the Pfam database (PF02701) against the grapevine genome database. Putative *Dof* genes were named according to Grimplet *et al.*^54^ using phylogenetic trees provided by Gramene^55^ (Figure 1). Grapevine Dof domains were aligned and all highly conserved residues described to the DNA-binding Dof domain were mapped in the 25 sequences (Supplementary Figure S1). All predicted gene models identified along with their chromosomal location and deduced protein length are presented in Figure 1. The putative functions of the grapevine Dof proteins were further investigated by predicting their subcellular localization using the Plant-mPLoc database.^45^ Only three sequences (VviDofL23, VviDofL24 and VviDofL25) not had their predicted subcellular location to the nucleus (Supplementary Table S3). Finally, intron and exon structures, as well as Dof domains, were determined and are displayed in Supplementary Figure S2 using FancyGene v1.4.^56^

**Figure 2:**
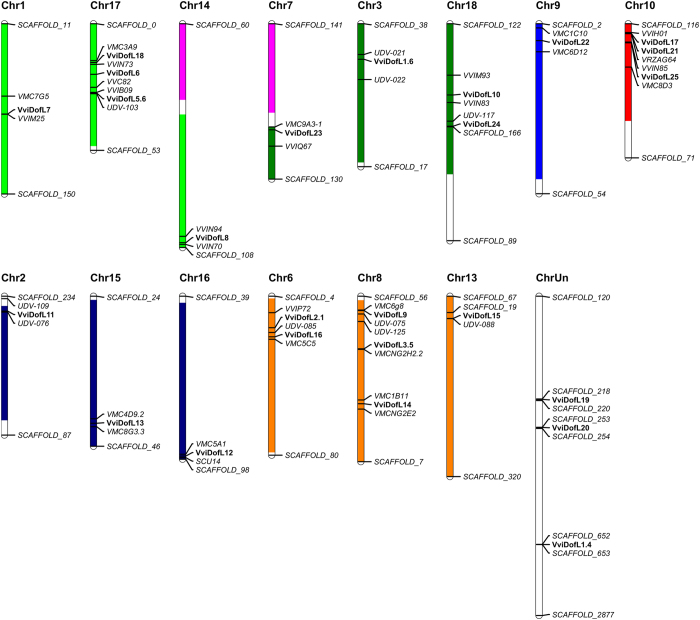
Chromosomal location of 25 *VviDofL* genes. Paralogous regions in the putative ancestral constituents of the grapevine genome are depicted in the color scheme following Jaillon *et al.*^40^ Molecular markers and scaffolds that help positioning genes are in italics.

The 25 putative *Dof* genes were found to be distributed in 14 out of the 19 grapevine chromosomes, as well as in the unplaced contigs chromosome (ChrUn), which contains sequences whose physical position on specific chromosomes have not yet been defined. As shown in Figure 2, chromosomes 8, 10 and 17 have three *DofL* genes each, chromosomes 6 and 18 both have two *DofL* genes and chromosomes 1, 2, 3, 7, 9, 13, 14, 15 and 16 have one *DofL* gene each. ChrUn also contains three *DofL* genes. Genome annotated scaffolds and molecular markers mapped near to *DofL* genes in each grapevine chromosome are also represented in Figure 2.

### Phylogenetic studies of predicted DofL proteins

The analysis of the deduced grapevine DofL protein sequences using the *MEME* software revealed the existence of several different motifs apart from the conserved Dof domain. Eleven motifs were identified with an *e*-value support lower than 1^−10^ and their annotation was performed using Pfam and SMART databases. However, only the Dof domain could be annotated (cyan motif in Figure 1 and in Supplementary Figure S3). The overall motif distribution of grapevine DofL sequences is shown in Figure 1. The Dof motif was the only one shared between all sequences. The other ten motifs were distributed unequally between DofL proteins (Figure 1). The consensus sequences of all motifs are shown in Supplementary Figure S3.

**Figure 3:**
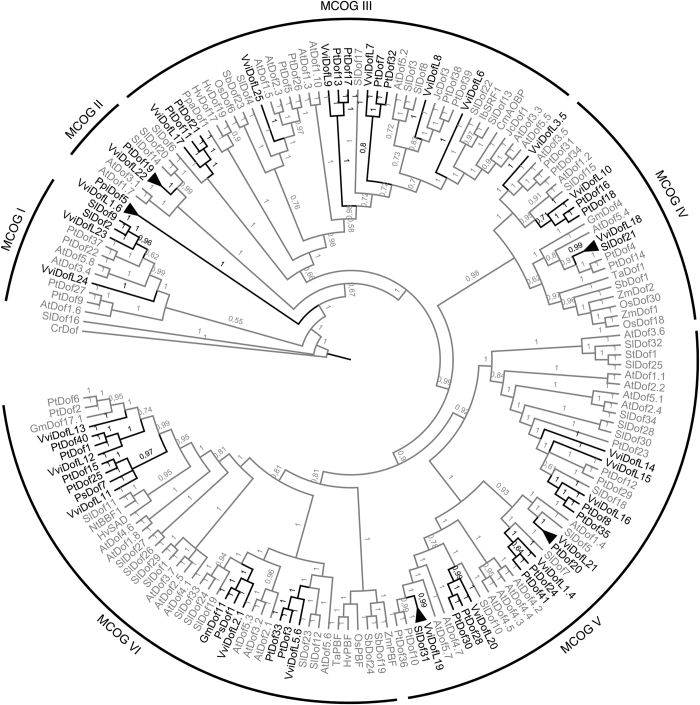
Phylogenetic tree of the full-length alignment of 167 Dof protein sequences. The resulting major clusters of orthologous genes (MCOGs) were numbered from I to VI. The tree was inferred using MrBayes v.3.2.6.^47^ Branch support is given by posteriori probability values shown next to the corresponding nodes (when>0.50). All accession codes used in the phylogenetic analysis are depicted in Supplementary Table S1. Clades comprising grapevine orthologs are collapsed; clades of grapevine proteins clusterized with paralogs or orthologs of other species are colored in black. At, *Arabidopsis thaliana*; Cm, *Cucurbita maxima*; Cr, *C*. *reinhardtii*; Gm, *Glycine max*; Hv, *Hordeum vulgare*; Ib, *Ipomoea batatas*; Jc, *J*. *curcas*; Nt, *Nicotiana tabacum*; Os, *Oriza sativa*; Ppa, *P. patens*; Ppi, *Pinus pinaster*; Ps, *Pisum sativum*; Pt, *Populus trichocarpa*; Sb, *Sorghum bicolor*; Sl, *Solanum lycopersicum*; St, *Solanum tuberosum*; Ta, *Triticum aestivum*; Vv, *V. vitifera*; Zm, *Zea mays*.

A phylogenetic study was carried out to evaluate evolutionary relationships within *DofL* genes. To this purpose, 167 sequences from 19 different plant species were used, including all identified sequences from Arabidopsis, grapevine, poplar and tomato, in addition to Dof proteins already described in barley, *C. reinhardtii*, *J. curcas*, maize, maritime pine, moss, pea, potato, pumpkin, rice, sorghum, soybean, sweet potato, tobacco and wheat (Supplementary Table S1). On the basis of previous phylogenetic analysis,^18,22,26^ the Dof sequence from *C. reinhardtii* was considered as a common Dof ancestor and was employed to root the tree. The phylogenetic tree generated by using full alignments produced high nodal support values (Figure 3). The tree topology obtained by the Bayesian Inference led to the identification of six major clusters of orthologous groups (MCOGs), numbered from I to VI. Interestingly, all MCOGs identified displayed at least one member from grapevine, poplar and tomato. Surprisingly, when curing the alignment, which converged to the Dof domain spanning region as the sole input sequence for the phylogenetic analysis, no trees with acceptable support values were produced (Supplementary Figure S4).

From the phylogenetic tree (Figure 3), 40 putative paralogs and 14 putative orthologs were identified among all 167 sequences analyzed. The majority of the paralogous pairs belonged to poplar (19) followed by Arabidopsis (10), tomato (9), barley and sorghum (one pair each). No paralogs were identified among grapevine sequences. Interestingly, eleven grapevine sequences formed clusters with paralogs from poplar (10) or tomato (1), namely VviDofL1.4, 5.6, 7, 9, 10, 11, 12, 16, 17, 20 and 23 (clades colored in black in Figure 3). Five putative orthologs were identified among grapevine: VviDofL1.6/PpiDof5, VviDofL22/PtDof19, VviDofL21/PtDof20, VviDofL18/SlDof21 and VviDofL19/SlDof31 (these clades are collapsed in Figure 3). Finally, VviDofL2.1 formed a cluster with the orthologous pair GmDof11/PsDof1.

### Expression analysis of *DofL *genes**

**Figure 4:**
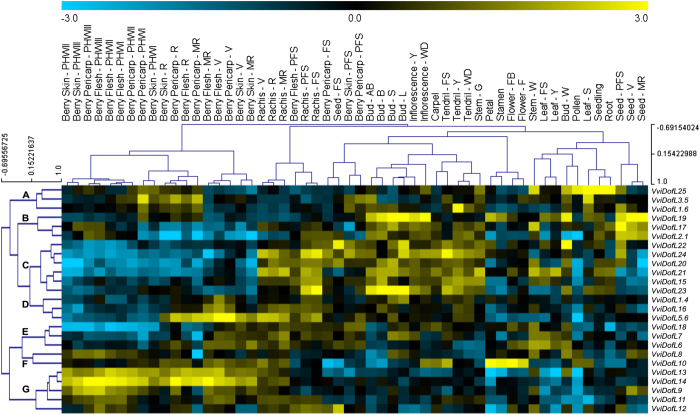
Transcript levels of *VviDofL* genes across 54 several grapevine cv Corvina tissues and organs. The color scheme used to represent expression level is yellow/blue: black boxes indicate low variation in gene expression, blue boxes indicate a fold decrease, and yellow boxes indicate a fold increase in relation to the mean value. Samples and genes were hierarchically clustered based on the average Pearson’s distance, resulting in seven major patterns of *VviDofL* mRNA levels (A–G). Abbreviations in the sample headline: for buds: AB, after-burst; B, bud burst; L, latent bud; S, bud swell; W, winter bud; for inflorescences: WD, well developed; Y, young; for flowers: F, flowering; FB, flowering begins; for tendrils: FS, mature; WD, well developed; Y, young; for leaves: FS, mature; S, senescing; Y, young; for berry pericarp, berry skin, berry flesh, seed and rachis: FS, fruit set; MR, mid-ripening; PFS, post-fruit set; PHWI, post-harvest withering (first month); PHWII, post-harvest withering (second month); PHWII, post-harvest withering (third month); R, ripening; V, véraison; for stems: G, green; W, woody. Data obtained as described in Fasoli *et al*.^49^

The expression profiles of putative grapevine *VviDofL* genes were retrieved from a global transcriptomic atlas comprising 54 tissues, organs or developmental stages.^49^ Overall, higher transcript accumulation was found in rachis, tendrils, buds and whole inflorescences, while low levels were identified in petals, stamens, pollen and berry skins. Hierarchical clustering analysis was carried out in order to find groups of *VviDofL* genes with similar transcript level profiles across samples. Groups were mostly composed of samples originated from the same tissue/organ (Figure 4). However, *VviDofL* expression patterns in buds, stems and berries were not totally clustered. A clear distinction could be observed between green/vegetative and mature/woody tissues and organs. In addition, senescing leaves presented marked differences in the patterns of *VviDofL* gene expression relatively to other leaf samples.

Seven major patterns of *VviDofL* mRNA accumulation were identified through samples (A–G in Figure 4). Genes from group A presented higher transcript levels in several berry stages (mainly ripening to mid-ripening). In addition,* VviDofL25* showed high expression in pollen, root, seedling and senescing leaves. Genes from group B were mainly induced in inflorescences, buds and seeds at the stages of post-fruit set to ripening. Interestingly, genes from group C showed low transcript amounts in all berry stages, except at the post-fruit set stage. Moreover, these genes were induced in tendril, rachis and bud stages. Transcripts from this group were presented in low levels in all four seed stages analyzed, with the exception of *VviDofL20*, *VviDofL22* and *VviDofL24* that were induced in seed at the fruit set and post-fruit set stages. Considering the three genes that integrate group D, only *VviDofL5.6* and *VviDofL16* were induced in rachis and berries from véraison to ripening stages, with *VviDofL5.6* showing the highest expression levels. Genes from group E and F showed minor expression variation in the samples analyzed. However, *VviDofL10* was highly induced in petals, stamens and flowers. Finally, genes from group G were mainly induced in berry stages (véraison to post-harvest withering III), with *VviDofL13* and *VviDofL14* showing the highest expression levels. In summary, a broad expression pattern of *VviDofL* genes was found across the 54 grapevine developmental stages analyzed.

**Figure 5:**
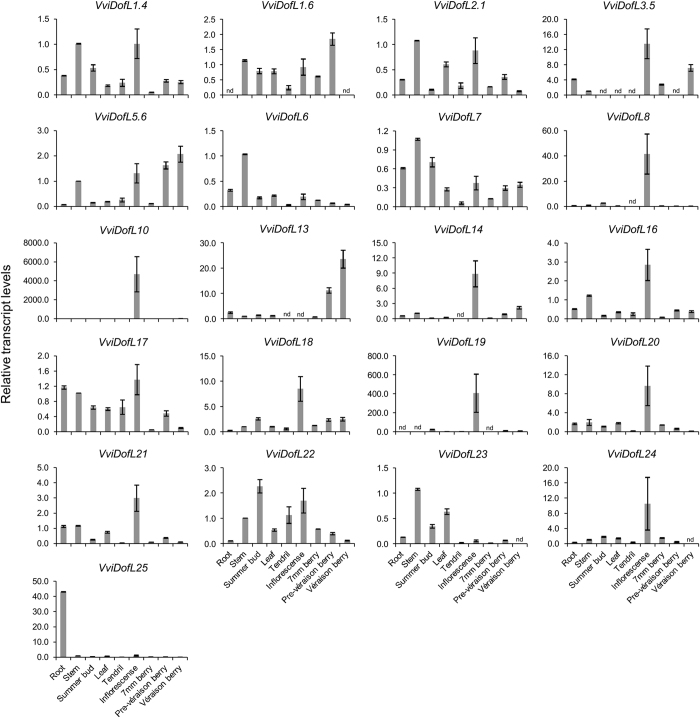
Expression profiles of *VviDofL* genes in nine grapevine cv Pinot Noir vegetative and reproductive organs by real-time PCR. Relative transcript levels in stems were set to 1, except *VviDofL19* whose leaf samples were set to 1. ND, non-detected. Standard error bars are shown.

In order to complement the whole-transcriptome data, steady-state mRNA levels of all *VviDofL* genes were investigated by real-time PCR in nine grapevine organs and tissues of the Pinot Noir grapevine variety. The overall grapevine *Dof* gene expression was quite diverse in these samples (Figure 5). Eleven *VviDofL* genes exhibited their highest expressions levels in inflorescences (*VviDofL3.5*, *VviDofL8*, *VviDofL10*, *VviDofL14*, *VviDofL16*, *VviDofL17*, *VviDofL18*, *VviDofL19*, *VviDofL20*, *VviDofL21*, and *VviDofL24*), three in stems (*VviDofL6*, *VviDofL7* and *VviDofL23*), two in stems and inflorescences (*VviDofL1.4* and *VviDofL2.1*), one gene in summer buds and inflorescences (*VviDofL22*), one in inflorescences, pre-véraison and véraison berries (*VviDofL5.6*), one in roots (*VviDofL25*), one in pre-véraison berries (*VviDofL1.6*), and one in véraison berries (*VviDofL13*). The genes with the highest expression specificity in the grapevine organs tested were *VviDofL6*, *VviDofL8*, *VviDofL10*, *VviDofL19* and *VviDofL25*. None of the *VviDofL* genes seemed to be specific to tendrils or leaves, or to have any pronounced level of mRNA accumulation in these organs. After several trials of reamplification, no measurable amplicons for *VviDofL9*, *VviDofL11*, *VviDofL12* and *VviDofL15* was obtained (Figure 5).

## Discussion

Velasco *et al.*^39^ identified 62 families of transcription factors in the consensus genome sequence of the heterozygous grapevine ‘Pinot Noir’ and, among them, Dof transcription factors. Corroborating those findings, we were able to identify, phylogenetically characterize, and describe the patterns of steady-state mRNA levels of 25 grapevine *Dof* genes. Among the seeded plant genomes annotated for *Dof* genes so far, grapevine, sugarcane and pine showed the smallest numbers.^21,22^ Although 25 putative *Dof* genes were found in grapevine and sugarcane,^33^ only 10 were identified in pine.^27,30^ Other species showed more members for this gene family, ranging from 26 in barley to 78 in soybean.^21,22,26,32^

In the present work, the four characteristic cysteine residues for zinc docking, as well as other well conserved sequences, were identified in all 25 grapevine DofL proteins, suggesting that their Dof domains are functional (Supplementary Figure S1). Additionally, the predicted physical localization of *DofL* genes in grapevine chromosomes was presented (Figure 2). In addition to the Dof domain, a plethora of other motifs is shared among DofL proteins (Figure 1). This finding was already observed in several other reports.^22,24–26,29–31^ The presence, absence or position of these motifs may affect the functional role of each Dof transcription factor. However, no similarity with known domains was found when comparing these sequences with the Pfam or SMART databases. Surprisingly, the comparison of the 10 grapevine non-Dof motifs identified (Supplementary Figure S3) with non-Dof motifs of other studies^22,24–26,29–31^ revealed similarities between the studies. This finding reinforces the need to conduct further investigations to unveil the functional roles of these still uncharacterized domains.

Comparative genomic studies are able to track characteristic features in multiple genomes. The identification of orthologous and paralogous sequences, along with functional information, can be useful tools to predict gene function.^57,58^ Moreover, gene duplication events are a common feature during genome and gene family evolution, and this seems to be the case in the *Dof* expansion. Moreno-Risueno *et al.*^26^ suggested that duplication events from an ancestral *Dof* gene would have triggered the expansion of this family, resulting in neo-, sub- and pseudogenization processes that likely lead to their high range of functions. Indeed, *Dof* genes are exclusive of the green lineage, increasing from a single-copy gene in *C. reinhardtii* to a multicopy family with numerous functions in seeded plants.^18^ In this scenario, the identification of paralogous and orthologous genes along with functional information about *Dof* family members can be used to infer gene function, although further investigations are required to confirm these predictions.

Dof sequences from grapevine, Arabidopsis, poplar and tomato, in addition to Dof proteins already described in other species, were compared using a phylogenetic approach with the aim of improving the knowledge about the function of the *VviDofL* genes (Figure 3). The results fit well with previous reports, given that our analysis rendered the same 22 Dof paralogous and orthologous pairs identified by Yang *et al.*,^25^ 11 identified by Yanagisawa^1^ and 7 identified by Cai *et al.*.^31^ Its worth to mention that several other pairs identified by these groups formed new paralogs or orthologs in our set of sequences. This finding can be explained by the large set of sequences used in our analysis. As one could expect in a comparative genome analysis, the number of orthologous sequences found between grapevine and poplar (12 pairs; Figure 3) was higher than the one found between poplar and rice (four pairs in Yang *et al.*^25^). Additionally, the strategy of comparing *Dof* genes from species with established functions yielded four pairs of orthologs besides the ones with poplar (Figure 3). These *Dof* genes were already characterized in maritime pine and tomato and may be an important resource to better understand the *VviDofL* functions in grapevine. Interestingly, grapevine sequences did not form paralogs suggesting that neofunctionalization events could be held accountable in the expansion of this gene family in grapevine. As several authors have previously observed,^24–26^ the Dof domain is extremely well conserved among proteins and, for phylogenetic comparisons, the only strategy that generate consistent trees was based on sequences outside the Dof domain. In accordance, curing of the protein alignment was not effective in producing better-structured trees given that the Bayesian algorithm was not able to branch the tree (Supplementary Figure S4). Lijavetzky *et al.*^24^ recognized that several clusters remained with poor supporting values in their phylogenetic analysis as the consequence of using only the conserved Dof domain (50 amino acids in length) in alignments.

Genome-wide studies describing the transcript accumulation patterns of *Dof* genes are found in the literature, reporting differential expression of *Dof* genes among tissues, organs or in response to abiotic stresses,^25,26,28–31,34–37,59^ but little information is available for grapevine *Dof* genes.^23^ In this work, by exploring genome-wide analysis by microarray in combination with real-time PCR, specific *VviDofL* transcriptional patterns were found. Transcriptome data obtained from various grapevine samples showed different *VviDofL* expression signatures according to the tissue/organ tested (Figure 4). Interestingly, some tissues/organs and mature/woody samples tended to display transcript accumulation profiles considerably distant from their vegetative/green counterparts. This trend was also observed for the whole set of organ-specific grapevine genes when screened for mRNA levels in the same samples.^49^ Furthermore, several expression profiles were obtained by real-time PCR (Figure 5) and support the microarray data. This finding is very interesting given that the plant material used in the two approaches is highly divergent, suggesting their involvement in similar processes regardless the cultivar. Finally, these results indicate that functional roles are being played by *VviDofL* genes during specific developmental stages, especially in reproductive tissues.

A recent review on the subject highlighted the importance of *Dof* genes during three main processes: metabolism regulation, seed development and tissue differentiation.^22^ The whole transcriptome data strongly suggest a clear relationship between *VviDofL* genes and the development of berries, seeds and flowers. Although most *VviDofL* genes presented low levels of transcripts during berry development (Figure 4), genes from group G, mainly *VviDofL13* and *VviDofL14*, were highly induced in berries from véraison to post-harvest withering III stages. In addition, *VviDofL3.5* (group A) displayed higher expression levels in berries at mid-ripening to ripening stages, and *VviDofL5.6* (group D) showed high transcript levels in berries from véraison to ripening stages. Similar results were obtained by real-time PCR for *VviDofL5.6* and *VviDofL13* (Figure 5). Costenaro-da-Silva *et al.*^23^ reported a *DofL* gene (*VviDofL18* in this work) being induced during berry developmental stages 2–6 weeks after fruit set; however, this relationship still lacks an in-depth characterization. Berry development in grapevine is a finely tuned complex process, and the results described in this work suggest that *VviDofL3.5, 5.6, 13* and *14* are important players in the regulation of berry development in grapevine. Complementarily, there is a widely accepted positive correlation between berry weight and seed content in grapevine segregating populations, possibly due to the action of growth regulators produced by seeds.^60^ In this respect, seeds can influence berry development, also having important roles in enological traits, given that seeds are one of the major providers of tannins during berry growth.^61^ Indeed, several *VviDofL* genes were highly induced in seed samples and they may be playing roles during seed development. This seems to be the case of genes from group B and genes *VviDofL20*, *VviDofL22* and *VviDofL24* from group C (Figure 4). These genes presented a remarkable accumulation of transcripts in all seed stages analyzed (fruit set to mid-ripening) and might be acting together in seed development. Interestingly, *VviDofL2.1* from group B, which was almost exclusively expressed in seeds from post-fruit set to mid-ripening stages (Figure 4), exhibited orthology with GmDof11 and PsDof1 (Figure 3). GmDof11, along with GmDof4, is involved in the regulation of genes associated with the synthesis of fatty acids, controlling the accumulation of lipids in soybean seeds.^59^ It is tempting to speculate that *GmDof11* and *VviDofL2.1* share conserved functions, although functional studies are needed to confirm or refute these statements. Furthermore, several functional studies reported the involvement of Dof proteins in the regulation of seed content, germination and/or development. Dof proteins from barley (HvSAD and HvPBF), maize (ZmPBF) and rice (OsPBF) were shown to activate several genes during seed development that are responsible for 15% of the total endosperm protein content.^10–14^ These Dof transcription factors also participate on the mobilization of storage compounds in order to provide nutrients for seed germination and seedling growth.^8^ In addition, DAG1 (Dof Affecting Germination 1) and DAG2 act on a maternal switch that is responsible for controlling seed germination in Arabidopsis.^3–6^ In this context, the transcriptional data gathered in this work suggest that several *VviDofL* genes are playing important roles during the course of berry and seed development.

Eleven *VviDofL* genes were mainly expressed in inflorescences when screened for mRNA levels by real-time PCR (Figure 5), and *VviDofL10*, *VviDofL19* and *VviDofL24* also presented flowering-specific transcription profiles in the whole transcriptome data (Figure 4). The identification of similar expression profiles between microarray and real-time PCR approaches, comparing samples from different cultivars, orchard management practices and climate growth conditions, strongly suggest that these genes have important regulatory roles during flowering that may be evolutionally conserved between grapevine varieties. In the literature, few reports about Dof functions during flowering are found,^19,20,62^ although some Dofs were described by their involvement in daylength regulation of flowering through interactions with CONSTANS.^15–18^ However, inside a subclade of MCOG V, VviDofL19 is very close to AtDof4.7 (Figure 3), which is involved in the control of floral abscission as part of a transcription complex that directly regulates the expression of cell wall hydrolysis enzymes.^19^ Moreover, ZmDof1 from maize was described as a transcriptional repressor of pollen development.^20^ Finally, flower-specific expression of *Dof* genes was already identified for *AtDof4.2*, which negatively and positively regulates the transcription of flavonoid and hydroxycinnamic acid production, respectively.^62^ Taken together, our findings strongly suggest that several VviDofLs might be acting in specific functions during flower development. Although promising, the genes herein identified still need further investigations in order to unveil their functional roles.

In conclusion, we identified the probable full set of *DofL* genes in grapevine. The phylogenetic analysis resulted in the identification of seven MCOGs that contains members from 18 plant species. Our results confirm that recurrent duplications and diversification from an original *Dof* ancestor led to the formation of this complex family of transcription factors specific to Viridiplantae. In view of important genome duplication events leading to gene redundancy in the history of plant diversification (see Jaillon *et al.*^40^ for example), a combination of phylogenetically inferred relationships with functional data will be essential to effectively establish conserved and diverged roles of the *DofL* gene family in grapevine and in other plant species. Finally, our expression data revealed the involvement of *VviDofL* genes mainly in berry, seed and flower development in grapevine. In this work, we gathered consistent results that turn the *VviDofL* genes into good candidates to better understand the regulatory network associated with flowering and berry development.
