# Supplementary figures and images for: Transcriptome analyses of the Dof-like gene family in grapevine reveal its involvement in berry, flower and seed development
Source: Hortic Res. 2016 Aug 31;3:16042–. doi: 10.1038/hortres.2016.42 (PMC5005469; doi:10.1038/hortres.2016.42)

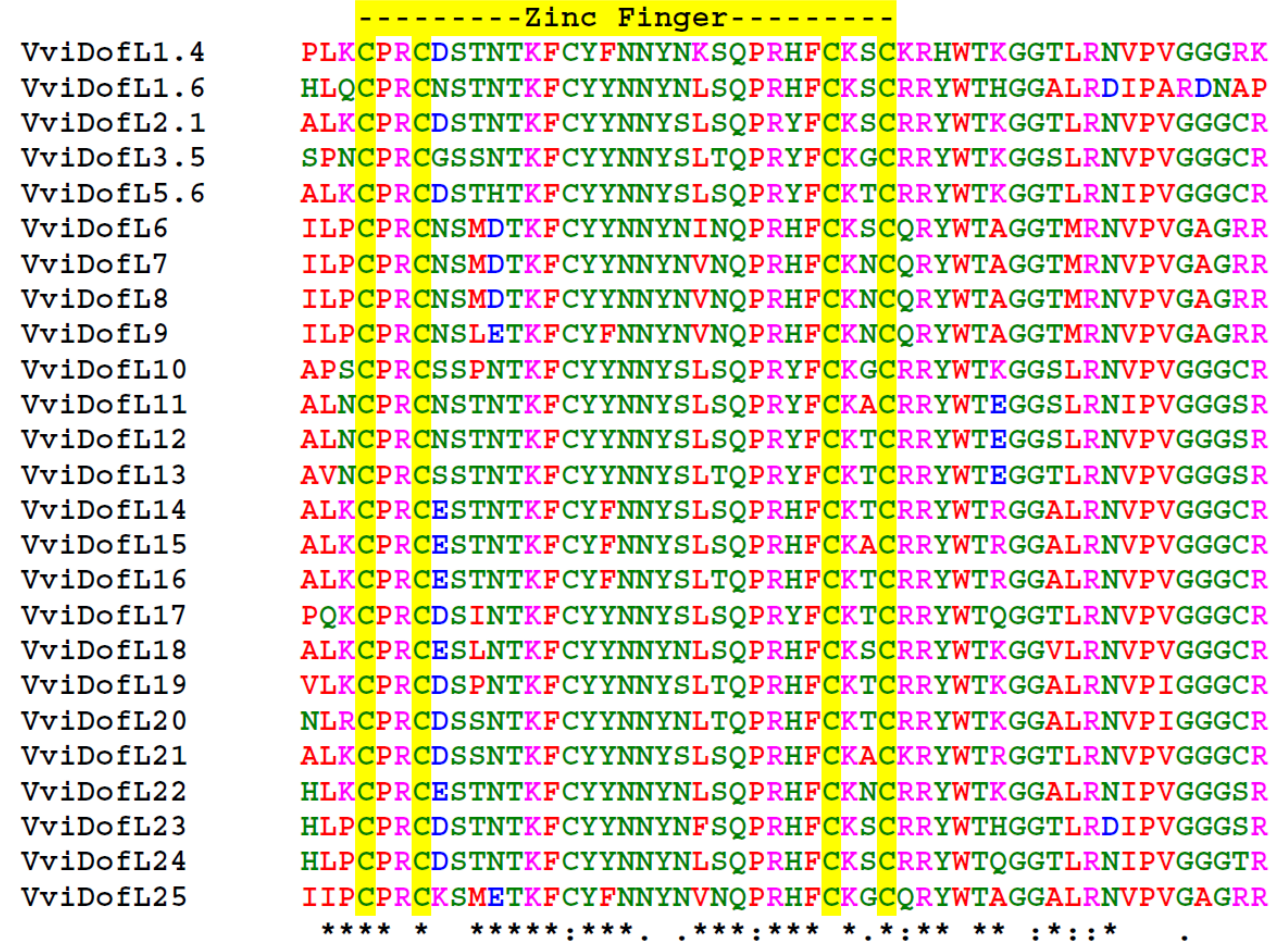

Supplement: Supplementary Figure S1 [file hortres201642-s1.jpg]

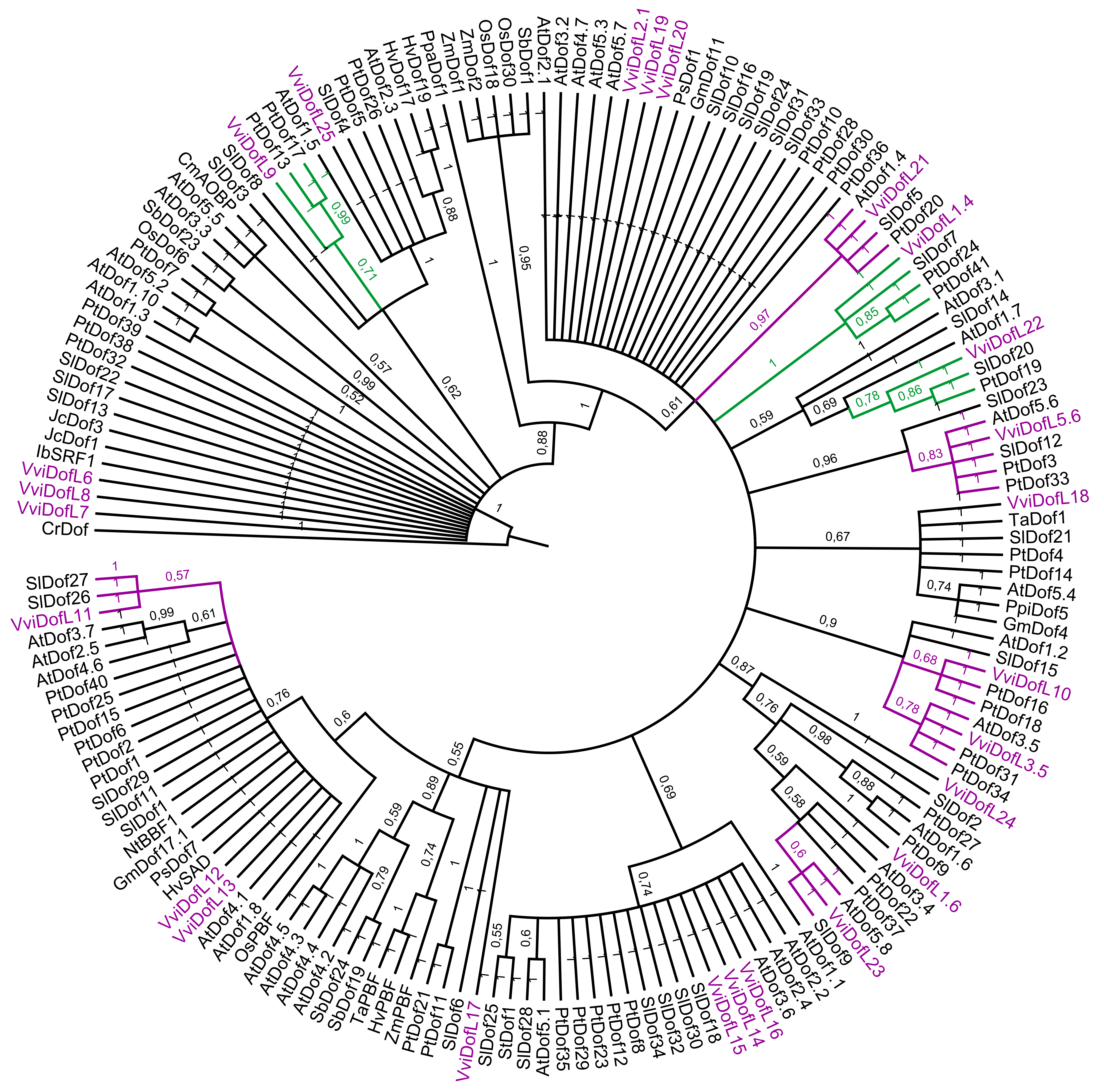

Supplement: Supplementary Figure S4 [file hortres201642-s4.jpg]
