# Supplementary material for: Transcriptome analyses of the Dof-like gene family in grapevine reveal its involvement in berry, flower and seed development
Source: Hortic Res. 2016 Aug 31;3:16042–. doi: 10.1038/hortres.2016.42 (PMC5005469; doi:10.1038/hortres.2016.42)
Supplement: Supplementary Tables S1–S3 [file hortres201642-s5.doc]

**Supplementary Table S1** Accession codes of *Dof* sequences used in the phylogenetic studies.

| **Gene name** | **Specie** | **Accession code** |
| --- | --- | --- |
| AtDof1.1 | *Arabidopsis thaliana* | At1g07640^a^ |
| AtDof1.2 | *Arabidopsis thaliana* | At1g21340^a^ |
| AtDof1.3 | *Arabidopsis thaliana* | At1g26790^a^ |
| AtDof1.4 | *Arabidopsis thaliana* | At1g28310^a^ |
| AtDof1.5 | *Arabidopsis thaliana* | At1g29160^a^ |
| AtDof1.6 | *Arabidopsis thaliana* | At1g47650^a^ |
| AtDof1.7 | *Arabidopsis thaliana* | At1g51700^a^ |
| AtDof1.8 | *Arabidopsis thaliana* | At1g64620^a^ |
| AtDof1.10 | *Arabidopsis thaliana* | At1g69570^a^ |
| AtDof2.1 | *Arabidopsis thaliana* | At2g28510^a^ |
| AtDof2.2 | *Arabidopsis thaliana* | At2g28810^a^ |
| AtDof2.3 | *Arabidopsis thaliana* | At2g34140^a^ |
| AtDof2.4 | *Arabidopsis thaliana* | At2g37590^a^ |
| AtDof2.5 | *Arabidopsis thaliana* | At2g46590^a^ |
| AtDof3.1 | *Arabidopsis thaliana* | At3g21270^a^ |
| AtDof3.2 | *Arabidopsis thaliana* | At3g45610^a^ |
| AtDof3.3 | *Arabidopsis thaliana* | At3g47500^a^ |
| AtDof3.4 | *Arabidopsis thaliana* | At3g50410^a^ |
| AtDof3.5 | *Arabidopsis thaliana* | At3g52440^a^ |
| AtDof3.6 | *Arabidopsis thaliana* | At3g55370^a^ |
| AtDof3.7 | *Arabidopsis thaliana* | At3g61850^a^ |
| AtDof4.1 | *Arabidopsis thaliana* | At4g00940^a^ |
| AtDof4.2 | *Arabidopsis thaliana* | At4g21030^a^ |
| AtDof4.3 | *Arabidopsis thaliana* | At4g21040^a^ |
| AtDof4.4 | *Arabidopsis thaliana* | At4g21050^a^ |
| AtDof4.5 | *Arabidopsis thaliana* | At4g21080^a^ |
| AtDof4.6 | *Arabidopsis thaliana* | At4g24060^a^ |
| AtDof4.7 | *Arabidopsis thaliana* | At4g38000^a^ |
| AtDof5.1 | *Arabidopsis thaliana* | At5g02460^a^ |
| AtDof5.2 | *Arabidopsis thaliana* | At5g39660^a^ |
| AtDof5.3 | *Arabidopsis thaliana* | At5g60200^a^ |
| AtDof5.4 | *Arabidopsis thaliana* | At5g60850^a^ |
| AtDof5.5 | *Arabidopsis thaliana* | At5g62430^a^ |
| AtDof5.6 | *Arabidopsis thaliana* | At5g62940^a^ |
| AtDof5.7 | *Arabidopsis thaliana* | At5g65590^a^ |
| AtDof5.8 | *Arabidopsis thaliana* | At5g66940^a^ |
| PtDof1 | *Populus trichocarpa* | estExt_fgenesh1_pg_v1.C_LG_XV0095^b^ |
| PtDof2 | *Populus trichocarpa* | fgenesh1_pg.C_LG_II001614^b^ |
| PtDof3 | *Populus trichocarpa* | eugene3.00120748^b^ |
| PtDof4 | *Populus trichocarpa* | eugene3.00120553^b^ |
| PtDof5 | *Populus trichocarpa* | grail3.0071006701^b^ |
| PtDof6 | *Populus trichocarpa* | grail3.0035031801^b^ |
| PtDof7 | *Populus trichocarpa* | eugene3.00101590^b^ |
| PtDof8 | *Populus trichocarpa* | estExt_Genewise1_v1.C_LG_IX4257^b^ |
| PtDof9 | *Populus trichocarpa* | estExt_Genewise1_v1.C_LG_II0357^b^ |
| PtDof10 | *Populus trichocarpa* | grail3.0027013501^b^ |
| PtDof11 | *Populus trichocarpa* | fgenesh1_pg.C_LG_IV000504^b^ |
| PtDof12 | *Populus trichocarpa* | grail3.0261000401^b^ |
| PtDof13 | *Populus trichocarpa* | eugene3.00190459^b^ |
| PtDof14 | *Populus trichocarpa* | eugene3.00150271^b^ |
| PtDof15 | *Populus trichocarpa* | estExt_Genewise1_v1.C_LG_III1128^b^ |
| PtDof16 | *Populus trichocarpa* | eugene3.00050960^b^ |
| PtDof17 | *Populus trichocarpa* | eugene3.00130645^b^ |
| PtDof18 | *Populus trichocarpa* | grail3.0003061201^b^ |
| PtDof19 | *Populus trichocarpa* | grail3.0044005601^b^ |
| PtDof20 | *Populus trichocarpa* | grail3.0124004901^b^ |
| PtDof21 | *Populus trichocarpa* | eugene3.01240063^b^ |
| PtDof22 | *Populus trichocarpa* | eugene3.00070983^b^ |
| PtDof23 | *Populus trichocarpa* | eugene3.00060710^b^ |
| PtDof24 | *Populus trichocarpa* | grail3.0093009501^b^ |
| PtDof25 | *Populus trichocarpa* | fgenesh1_pg.C_LG_I000728^b^ |
| PtDof26 | *Populus trichocarpa* | eugene3.00040392^b^ |
| PtDof27 | *Populus trichocarpa* | estExt_Genewise1_v1.C_400561^b^ |
| PtDof28 | *Populus trichocarpa* | fgenesh1_pg.C_LG_VII000931^b^ |
| PtDof29 | *Populus trichocarpa* | estExt_fgenesh1_pg_v1.C_LG_VIII1519^b^ |
| PtDof30 | *Populus trichocarpa* | eugene3.00050449^b^ |
| PtDof31 | *Populus trichocarpa* | fgenesh1_pg.C_LG_XVI000552^b^ |
| PtDof32 | *Populus trichocarpa* | fgenesh1_pm.C_LG_VIII000324^b^ |
| PtDof33 | *Populus trichocarpa* | eugene3.00150562^b^ |
| PtDof34 | *Populus trichocarpa* | grail3.0030001801^b^ |
| PtDof35 | *Populus trichocarpa* | fgenesh1_pg.C_LG_I001755^b^ |
| PtDof36 | *Populus trichocarpa* | grail3.0089006501^b^ |
| PtDof37 | *Populus trichocarpa* | fgenesh1_pm.C_LG_V000172^b^ |
| PtDof38 | *Populus trichocarpa* | estExt_fgenesh1_pg_v1.C_LG_IV1063^b^ |
| PtDof39 | *Populus trichocarpa* | estExt_fgenesh1_pg_v1.C_LG_XVII0409^b^ |
| PtDof40 | *Populus trichocarpa* | grail3.0031019801^b^ |
| PtDof41 | *Populus trichocarpa* | grail3.0091000801^b^ |
| SlDof1 | *Solanum lycopersicum* | Solyc01g096120^c^ |
| SlDof2 | *Solanum lycopersicum* | Solyc02g065290^c^ |
| SlDof3 | *Solanum lycopersicum* | Solyc02g067230^c^ |
| SlDof4 | *Solanum lycopersicum* | Solyc02g076850^c^ |
| SlDof5 | *Solanum lycopersicum* | Solyc02g077950^c^ |
| SlDof6 | *Solanum lycopersicum* | Solyc02g077960^c^ |
| SlDof7 | *Solanum lycopersicum* | Solyc02g078620^c^ |
| SlDof8 | *Solanum lycopersicum* | Solyc02g088070^c^ |
| SlDof9 | *Solanum lycopersicum* | Solyc02g090220^c^ |
| SlDof10 | *Solanum lycopersicum* | Solyc02g090310^c^ |
| SlDof11 | *Solanum lycopersicum* | Solyc03g082840^c^ |
| SlDof12 | *Solanum lycopersicum* | Solyc03g112930^c^ |
| SlDof13 | *Solanum lycopersicum* | Solyc03g115940^c^ |
| SlDof14 | *Solanum lycopersicum* | Solyc03g121400^c^ |
| SlDof15 | *Solanum lycopersicum* | Solyc04g070960^c^ |
| SlDof16 | *Solanum lycopersicum* | Solyc04g079570^c^ |
| SlDof17 | *Solanum lycopersicum* | Solyc05g007880^c^ |
| SlDof18 | *Solanum lycopersicum* | Solyc05g054510^c^ |
| SlDof19 | *Solanum lycopersicum* | Solyc06g005130^c^ |
| SlDof20 | *Solanum lycopersicum* | Solyc06g062520^c^ |
| SlDof21 | *Solanum lycopersicum* | SGN-U582777^d^ |
| SlDof22 | *Solanum lycopersicum* | Solyc06g069760^c^ |
| SlDof23 | *Solanum lycopersicum* | Solyc06g071480^c^ |
| SlDof24 | *Solanum lycopersicum* | Solyc06g075370^c^ |
| SlDof25 | *Solanum lycopersicum* | Solyc06g076030^c^ |
| SlDof26 | *Solanum lycopersicum* | Solyc08g008500^c^ |
| SlDof27 | *Solanum lycopersicum* | Solyc08g082910^c^ |
| SlDof28 | *Solanum lycopersicum* | Solyc09g010680^c^ |
| SlDof29 | *Solanum lycopersicum* | Solyc10g009360^c^ |
| SlDof30 | *Solanum lycopersicum* | Solyc10g086440^c^ |
| SlDof31 | *Solanum lycopersicum* | Solyc11g010940^c^ |
| SlDof32 | *Solanum lycopersicum* | Solyc11g066050^c^ |
| SlDof33 | *Solanum lycopersicum* | Solyc11g072500^c^ |
| SlDof34 | *Solanum lycopersicum* | Solyc00g024680^c^ |
| *VviDofL1.4* | *Vitis vinifera* | VIT_00s0652g00010^e^ |
| *VviDofL1.6* | *Vitis vinifera* | VIT_03s0063g01350^e^ |
| *VviDofL2.1* | *Vitis vinifera* | VIT_06s0004g03420^e^ |
| *VviDofL3.5* | *Vitis vinifera* | VIT_08s0105g00170^e^ |
| *VviDofL5.6* | *Vitis vinifera* | VIT_17s0000g08290^e^ |
| *VviDofL6* | *Vitis vinifera* | VIT_17s0000g06310^e^ |
| *VviDofL7* | *Vitis vinifera* | VIT_01s0026g02580^e^ |
| *VviDofL8* | *Vitis vinifera* | VIT_14s0108g00980^e^ |
| *VviDofL9* | *Vitis vinifera* | VIT_08s0056g01230^e^ |
| *VviDofL10* | *Vitis vinifera* | VIT_18s0001g15730^e^ |
| *VviDofL11* | *Vitis vinifera* | VIT_02s0025g02250^e^ |
| *VviDofL12* | *Vitis vinifera* | VIT_16s0098g01420^e^ |
| *VviDofL13* | *Vitis vinifera* | VIT_15s0046g00150^e^ |
| *VviDofL14* | *Vitis vinifera* | VIT_08s0007g00180^e^ |
| *VviDofL15* | *Vitis vinifera* | VIT_13s0019g01410^e^ |
| *VviDofL16* | *Vitis vinifera* | VIT_06s0004g04520^e^ |
| *VviDofL17* | *Vitis vinifera* | VIT_10s0003g00030^e^ |
| *VviDofL18* | *Vitis vinifera* | VIT_17s0000g04850^e^ |
| *VviDofL19* | *Vitis vinifera* | VIT_00s0218g00040^e^ |
| *VviDofL20* | *Vitis vinifera* | VIT_00s0253g00060^e^ |
| *VviDofL21* | *Vitis vinifera* | VIT_10s0003g00040^e^ |
| *VviDofL22* | *Vitis vinifera* | VIT_09s0002g02490^e^ |
| *VviDofL23* | *Vitis vinifera* | VIT_07s0255g00020^e^ |
| *VviDofL24* | *Vitis vinifera* | VIT_18s0001g11310^e^ |
| *VviDofL25* | *Vitis vinifera* | VIT_10s0003g01260^e^ |
| CmAOBP | *Curcubita maxima* | BAA08094^f^ |
| CrDof | *Chlamydomonas reinhardtii* | XP_001696918^f^ |
| GmDof4 | *Glycine max* | ABI16005^f^ |
| GmDof11 | *Glycine max* | ABI16012^f^ |
| GmDof17-1 | *Glycine max* | NP_001236720 |
| HvDof17 | *Hordeum vulgare* | AJ969263^f^ |
| HvDof19 | *Hordeum vulgare* | CAC85947^f^ |
| HvPBF | *Hordeum vulgare* | CAA04440^f^ |
| HvSAD | *Hordeum vulgare* | CAC85739^f^ |
| IbSRF1 | *Ipomea batatas* | AB469355^f^ |
| JcDof1 | *Jatropha curcas* | ACU80550^f^ |
| JcDof3 | *Jatropha curcas* | DQ109673^f^ |
| NtBBF1 | *Nicotiana tabacum* | CAA08755^f^ |
| OsDof6 | *Oryza sativa* | LOC_Os03g07360^g^ |
| OsDof18 | *Oryza sativa* | LOC_Os08g38220^g^ |
| OsDof30 | *Oryza sativa* | LOC_Os09g29960^g^ |
| OsPBF | *Oryza sativa* | BAA78574^f^ |
| PpaDof1 | *Physcomitrella patens* | BAL46025^f^ |
| PpiDof5 | *Pinus pinaster* | AM884254^f^ |
| PsDof1 | *Pisum sativum* | AB026297^f^ |
| PsDof7 | *Pisum sativum* | BAC81664^f^ |
| SbDof1 | *Sorghum bicolor* | AEF32402^f^ |
| SbDof19 | *Sorghum bicolor* | AEF32403^f^ |
| SbDof23 | *Sorghum bicolor* | AEF32404^f^ |
| SbDof24 | *Sorghum bicolor* | AEF32405^f^ |
| StDof1 | *Solanum tuberosum* | CAB89831^f^ |
| TaDof1 | *Triticum aestivum* | AAX54942^f^ |
| TaPBF | *Triticum aestivum* | AAS19857^f^ |
| ZmDof1 | *Zea mays* | CAA46875^f^ |
| ZmDof2 | *Zea mays* | CAA56287^f^ |
| ZmPBF | *Zea mays* | NP_001105400^f^ |

^a^ Accession code obtained from http://www.arabidopsis.org/

^b^ Accession code obtained from http://genome.jgi-psf.org/Poptr1/Poptr1.home.html

^c^ Accession code obtained from http://www.phytozome.net/

^d^ Accession code obtained from http://solgenomics.net/

^e^ Accession code obtained from http://genomes.cribi.unipd.it/

^f^ Accession code obtained from http://www.ncbi.nlm.nih.gov/

^g^ Accession code obtained from http://drtf.cbi.pku.edu.cn/index.php

| **Supplementary Table S2.** Primers employed in real-time PCR studies. | | |
| --- | --- | --- |
| **Gene Name** | **Forward primer** | **Reverse primer** |
| *VviDofL1.4* | CCCGATCCAAAGGCTAGAAT | ACTACCACCCTGCAACAAGG |
| *VviDofL1.6* | TCTGGGAGTTTCACCAGGTT | AACATGAATATCCGGCCAAG |
| *VviDofL2.1* | CAAACTGGTGGGCAGTTAGG | TGGTCCTCAGAGCATCAAGA |
| *VviDofL3.5* | CTCCGCCACTACCTCTTCTG | AGCTGGAAAGATGGATGGAA |
| *VviDofL5.6* | CATCCACCCGAGTCTCTCTC | TCTCCGGAAGGTGAAGAAGA |
| *VviDofL6* | TCAGCTCAGGTGATGTTGGA | GGGTTCTCATTGCTCTCTGC |
| *VviDofL7* | TCCAACGTCGTCTTCTCCTA | TGGGATCGTCAATCCTCAGT |
| *VviDofL8* | TTAGCGGGGGCAGACTCTTT | TTGAGCACCATGCTCTTCCT |
| *VviDofL9* | GTGCTGAGATGCGAACTGTC | GAGGAACAGGGTAACAGGGC |
| *VviDofL10* | GATCGACTTGGCTGTGGTTT | CACTCCATCGCCATGTCTTC |
| *VviDofL11* | GCCGCTCAAGATTTCAGAAC | CAAGCCCATCCAGAGAAAAA |
| *VviDofL12* | CTCGTCGGACGGTAACATTT | AAGAAATCCCGCGGTGATC |
| *VviDofL13* | TCTTCATCCTCATTCAAAAC | TTCTCGATTTTGGGCACTTC |
| *VviDofL14* | CTTCCATGCCATCCTATCTT | TAGAACCTGGCCTGATGGAG |
| *VviDofL15* | TTCTCATCCGTTCCGGTTTA | AGGCCTGATTGTGCCAGTA |
| *VviDofL16* | AACCATGGTTTTCTCATCTGTT | ATCAGCCATCGAACCAGGTC |
| *VviDofL17* | GCCGTGGGGCCAGCTCTGAG | CTGGTTGATTGAAGGGCTGT |
| *VviDofL18* | TAGCTTTCCCACCTCCTCCT | GCAGAGGAAACCCAGATGAG |
| *VviDofL19* | TGCCGGAAAAACAAGAAGAT | TGAATTGCACCTCCAAATCC |
| *VviDofL20* | GGGGTTGCCGGAAGAACAAG | CTGGGTGGCTCTCAGTAAGG |
| *VviDofL21* | CCTTTGAGAAAGGAAATGTTGG | GGAGGAATCACAACGAGGAC |
| *VviDofL22* | AGATGATGAGAATGAAGAAG | CTAATGGCCTTGTGGAAATAT |
| *VviDofL23* | GVGGTGGCACTGCTCTAGTG | AAAAGGCAGTGAGATTCAAAGG |
| *VviDofL24* | TTCTCTCATACATTCTTCTC | ACCCACTTGTTGGGGTCTC |
| *VviDofL25* | GAAAAGTAAGCCGCCCTGT | CTGCTCCTACGCCTCTTAGC |

**Supplementary Table S3.** Predicted subcellular localization using the Plant-mPloc database.^45^

| **Gene name** | **Predicted subcellular localization** |
| --- | --- |
| *VviDofL1.4* | Nucleus |
| *VviDofL1.6* | Nucleus |
| *VviDofL2.1* | Nucleus |
| *VviDofL3.5* | Nucleus |
| *VviDofL5.6* | Nucleus |
| *VviDofL6* | Nucleus |
| *VviDofL7* | Nucleus |
| *VviDofL8* | Nucleus |
| *VviDofL9* | Nucleus |
| *VviDofL10* | Nucleus |
| *VviDofL11* | Nucleus |
| *VviDofL12* | Nucleus |
| *VviDofL13* | Nucleus |
| *VviDofL14* | Nucleus |
| *VviDofL15* | Nucleus |
| *VviDofL16* | Nucleus |
| *VviDofL17* | Nucleus |
| *VviDofL18* | Nucleus |
| *VviDofL19* | Nucleus |
| *VviDofL20* | Nucleus |
| *VviDofL21* | Nucleus |
| *VviDofL22* | Nucleus |
| *VviDofL23* | Cell wall |
| *VviDofL24* | Cell wall |
| *VviDofL25* | Cytoplasm |
